# Pediatric incarcerated inguinal hernia: Traditional open or laparoscopic-assisted approach?

**DOI:** 10.1097/MD.0000000000037810

**Published:** 2024-05-03

**Authors:** Guofeng Zhang, Huanfei Ding, Di Wang, Fei Guo, Bowen Hu

**Affiliations:** a Department of Pediatric Surgery, The First Affiliated Hospital of Zhengzhou University, Zhengzhou, China; b Department of Ultrasound, The First Affiliated Hospital of Zhengzhou University, Zhengzhou, China; c Department of Hepatobiliary and Pancreatic Surgery, The First Affiliated Hospital of Zhengzhou University, Zhengzhou, China.

**Keywords:** inguinal hernia, laparoscopic surgery, open surgery, pediatric patients

## Abstract

The objective of this study was to compare the safety and efficacy of laparoscopic-assisted surgery and traditional open surgery for pediatric incarcerated inguinal hernia. A total of 58 pediatric patients with indirect incarcerated inguinal hernia between January 2014 and January 2020 were included in this study. The patients were divided into 2 groups; observational group who underwent laparoscopic-assisted surgery (n = 36), and a control group who underwent traditional open surgery (n = 22). The overall operation time, intraoperative blood loss, postoperative recovery time, length of hospital stay, occurrence of postoperative scrotal or vulvar hematomas, incidence of postoperative surgical site infection, and hernia recurrence were analyzed and compared between the 2 groups. Compared with the control group, the operation time (38.28 ± 5.90) minutes, intraoperative blood loss (1.15 ± 0.54 mL), postoperative recovery time (8.39 ± 1.42 h), and length of hospital stay (1.64 ± 0.59) were significantly lower in the observational group (*P *< .05). There was no incidence of scrotal or vulvar hematoma or surgical site infection in the observation group, which was significantly lower than that in the control group (*P *< .05). However, no statistically significant difference was found in the rate of postoperative hernia recurrence between the 2 groups (*P *> .05). In conclusion, laparoscopic-assisted surgery appears to be a safe and effective alternative approach to traditional open surgery for the treatment of pediatric incarcerated inguinal hernia. Its advantages include reduced trauma, faster recovery, shorter hospital stays, and fewer complications.

## 1. Introduction

Incarcerated inguinal hernia is frequently seen in the pediatric patients and is one of the most common surgical emergency. in the pediatric population.^[1–3]^ An incarcerated oblique inguinal hernia is a prevalent acute abdominal complication in pediatric surgery, with a tendency to cause serious complications in the absence of correct and timely treatment.^[4,5]^ In the past several decades, open surgical incision repair remains the only standard of care for the management of incarcerated indirect inguinal hernia. However, with the introduction and popularity of minimally invasive techniques in pediatric surgery, laparoscopic surgery has become the treatment of choice for incarcerated indirect inguinal hernia wherever resources and experiences are available.^[6,7]^

Although many studies have discussed the role of open surgery or laparoscopic surgery separately for the management of inguinal hernia in the pediatric patients.^[8,9]^ To date, there is no comparative study between open or laparoscopic surgery available for the treatment of incarcerated inguinal hernia. In this study, we retrospectively analyzed the clinical data of all patients who underwent either traditional open surgery or laparoscopic-assisted surgery for the treatment of incarcerated indirect inguinal hernias in children. We compared both groups with regards to overall procedure time, intraoperative blood loss, postoperative recovery time, duration hospital stay, postoperative complications rate, and hernia recurrence to evaluate the safety and efficacy of laparoscopic-assisted surgery versus traditional open surgery.

## 2. Materials and methods

### 2.1. Patients’ data and study protocol

This is a single center retrospective study of consecutive patients with incarcerated indirect inguinal hernia who underwent either traditional open surgery or laparoscopic-assisted surgery treatment at the First Affiliated Hospital of Zhengzhou University between January 2014 to January 2020. The study was approved by the institutional review board of the First Affiliated Hospital of Zhengzhou University. Informed consent was obtained from all the patients guardian before the procedure. The patients were divided into 2 groups: an observational group in which patients underwent laparoscopic surgery and a control group in which patients underwent traditional open surgery via inguinal incision. All operations were performed by the expert surgeons, with an experience of more than 500 laparoscopic and open surgeries.

### 2.2. Inclusion or exclusion criteria

The inclusion criteria were as followed: incarcerated indirect inguinal hernia diagnosed by ultrasonography, failure of manual reduction of the hernia, and stable vital signs without any indications of severe dehydration, sepsis, or abdominal distention. Exclusion criteria were as followed: severe hepatic and renal insufficiency, coagulation disorders, and ipsilateral cryptorchidism.

### 2.3. Description of the procedures

#### 2.3..1. Laparoscopic-assisted surgery

**Figure 1.**
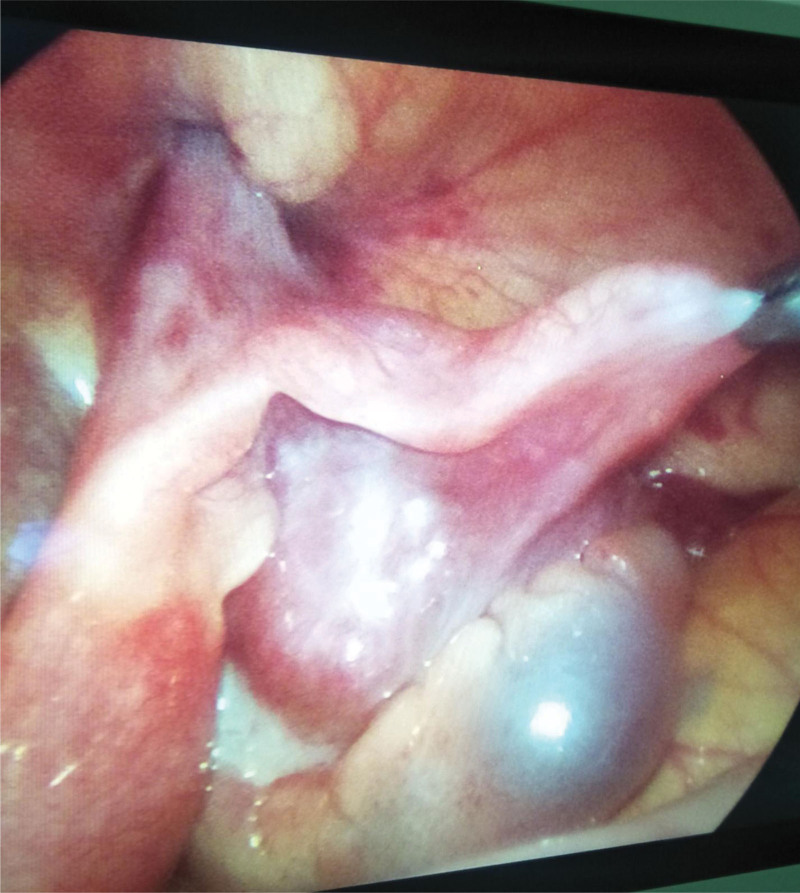
Traction of ovary with auxiliary forceps for the observation of incarcerated hernia contents.

**Figure 2.**
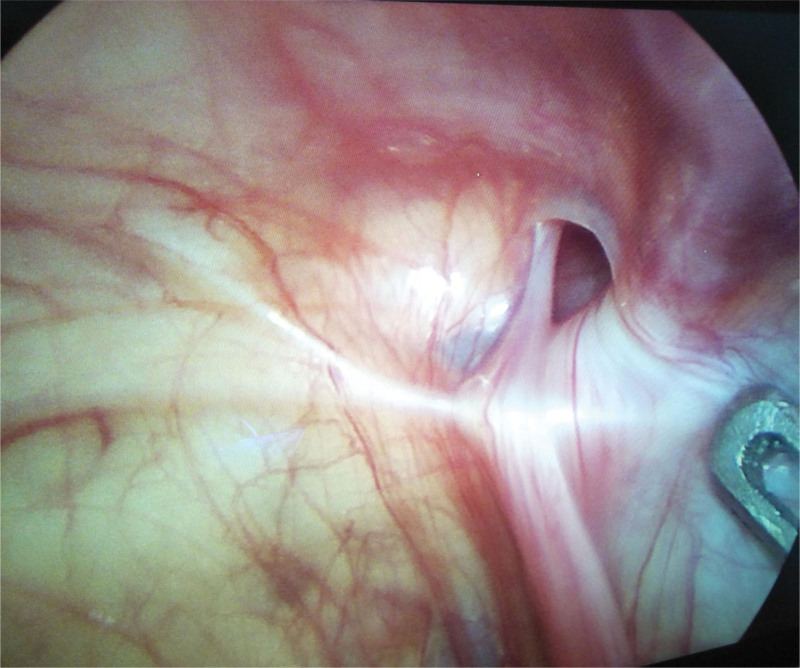
Intraoperative exploration of patent contralateral processus vaginalis.

**Figure 3.**
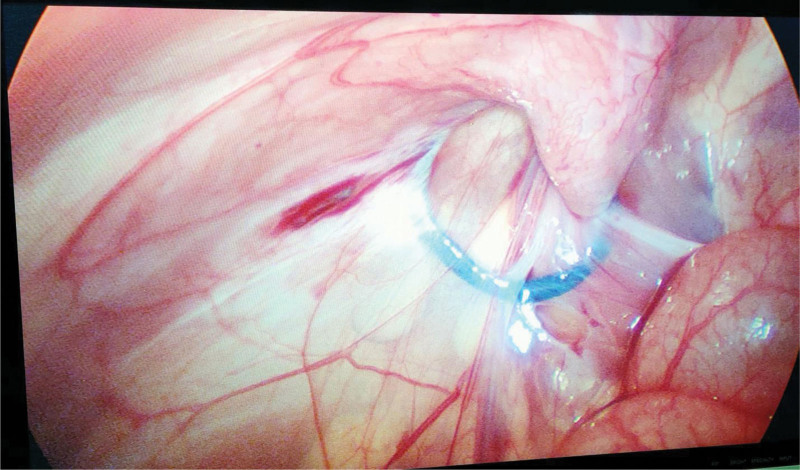
Confirming that the vas deferens and spermatic vessels lie just lateral to the thread.

All the patients in the observation group underwent laparoscopic-assisted incarcerated hernia reduction and high ligation of the hernial sac. The procedures were performed under general anesthesia. The patient was placed in the supine position with the buttocks slightly elevated. A 5 mm trocar was inserted through the umbilicus, and carbon dioxide pneumoperitoneum was established with pneumoperitoneum pressure maintained at 6 to 8 mm Hg. A laparoscopic light source was inserted and the contents of the incarcerated hernia were explored. Under direct laparoscopic visualization, the contents of the incarcerated hernia were meticulously reduced in size. In cases where hernia reduction was difficult, a 3 mm trocar was inserted into the lateral border of the rectus abdominis muscle, through which auxiliary forceps were passed to assist in the reduction. After successful reduction of the hernia contents, blood supply to the incarcerated hernia contents was observed (Fig. 1). In cases of intestinal necrosis, the umbilical incision was enlarged to facilitate extraction of the necrotic bowel to perform resection and intestinal anastomosis as necessary. Laparoscopic oophorectomy of the necrotic ovary was performed in cases of ovarian necrosis. Children who had relatively poor bowel or ovarian blood supply were observed for a duration of 10 minutes after hernia reduction, and the contralateral inguinal abdominal ring was explored simultaneously to verify the patency of the processus vaginalis peritonei (Fig. 2). The body surface projection of the deep inguinal ring on the affected side was located 1 to 2 cm above the midpoint of the inguinal ligament. An abdominal wall suture apparatus containing suture thread was inserted into the extraperitoneal space, and sutures were started from the inner half-circle of the deep ring, continued across the vas deferens, and ended in the outer half of the deep ring. The hernia sac was squeezed to drain the gas and fluid within the cavity while tightening the suture thread to tie a knot into the extraperitoneal space (Fig. 3).

#### 2.3..2. Traditional open surgery

The control group patients underwent traditional inguinal incision surgery. A transverse incision of approximately 2 to 3 cm was made through the inguinal crease on the affected side. The skin was incised open, subcutaneous tissue was separated, the hernial sac was located and incised, and its contents were inspected for ischemic necrosis. If no ischemic necrosis was detected, the contents of the hernia were returned to the abdominal cavity. However, if ischemic necrosis was present, resection of the necrotic bowel and subsequent intestinal anastomosis was performed. Similarly, if testicular necrosis was found, a combined resection was performed. In cases where retrieving the contents within the hernia was difficult, the orifice of the inner ring had to be opened, and the aponeurosis of the transverse abdominis was cut if necessary. Upon completion of retrieval and replacement of the hernia contents, the empty hernial sac was removed and ligated at a higher level. Following the reconstruction of the inner ring, the subcutaneous and skin layers were sutured closed.

### 2.4. Observation indicators

The operation time, intraoperative blood loss, postoperative recovery time, length of hospital stay, occurrence of postoperative scrotal or vulvar hematoma, incidence of postoperative surgical site infection, and hernia recurrence were compared between the 2 groups. All children included in the study were followed up at the outpatient clinic for 1 year after the completion of surgery.

### 2.5. Statistical analysis

Statistical package for the social sciences 20.0 statistical software were performed using statistical analysis of statistical package for the social sciences data. Normally distributed data are expressed as $\bar{x}\;\pm s$. Comparisons between groups with equal variance were carried out using the independent samples t-test, and the counting data were analyzed using the χ^2^ test. *P *< .05.

## 3. Results

The observational group included 36 patients (28 males and 8 females), with a mean age of 1.30 ± 0.72 years, 25 cases occurred on the right side and 11 cases on the left side. The incarceration time was < 12 hours in 6 cases, 12 to 24 hours in 29 cases, and > 24 hours in 1 case. The control group included 22 patients (16 males and 6 females) with a mean age of 1.45 ± 0.72 years, 15 cases occurred on the right side, and 7 cases occurred on the left side. The incarceration time was < 12 hours in 4 cases, and 12 to 24 hours in 18 cases. There were no statistically significant differences in sex, age, location of incarceration, or incarceration time between the 2 groups (*P* > .05). The baseline characteristics of the included patients are summarized in Table 1.

**Table 1:** Baseline characteristics of all the patients.

Group	Gender		Age (yr)	Incarceration location		Incarceration time (h)
	Male	Female		Right side	Left side	
Observation	28	8	1.30 ± 0.72	25	11	15.09 ± 6.16
Control	16	6	1.45 ± 0.72	15	7	16.31 ± 6.95
t/χ^2^	0.1902		0.7699	0.0249		0.6971
*P* value	.6627		.4446	.08745		.4887

Compared with control group, the operation time (38.28 ± 5.90) minutes, intraoperative blood loss (1.15 ± 0.54) mL, postoperative recovery time (8.39 ± 1.42) hours, and length of hospital stay (1.64 ± 0.59) days, was significantly lower in the observational group (*P* < .05). Comparisons of postoperative indicators between the 2 groups are summarized in Table 2.

**Table 2:** Comparison of postoperative related indicators between the 2 groups.

Group		Operation time (min)	Intraoperative bleeding (mL)	Postoperative recovery time (h)	Length of stay (d)
Observation	36	38.28 ± 5.90	1.15 ± 0.54	8.39 ± 1.42	1.64 ± 0.59
Control	22	57.09 ± 5.34	3.23 ± 0.75	18.77 ± 3.31	4.18 ± 1.10
t value		12.202	12.2578	16.5542	11.4557
*P* value		<.05	<.05	<.05	<.05

There was no incidence of scrotal or vulvar hematoma, and the incidence of surgical site infection in the observation group, which was significantly lower than that in the control group (*P* < .05). The observation group had no cases of postoperative recurrence, whereas the control group had 1 case of hernia recurrence. There were no statistically significant differences between the 2 groups (*P* > .05). A comparison of the postoperative complications and recurrence between the 2 groups is summarized in Table 3. All the patients were followed by telephone. During the 12 to 48 months of follow up no postoperative morbidities were noted.

**Table 3:** Comparison of postoperative complications and recurrence between the 2 groups.

Group	Surgical site infection (cases)	Scrotal or vulvar hematoma (cases)	Recurrence (cases)
Observation	0	0	0
Control	3	3	1
χ^2^	5.0876	5.0876	1.5652
*P*	<.05	<.05	.2109

## 4. Discussion

The majority of inguinal hernias in children are indirect hernias caused by patent processus vaginalis.^[9]^ One of the 3 cases occur before the age of 6 months, and the incidence in males is almost 5 to 10 times compared to females.^[10,11]^ Incarceration may occur in 1/6 of indirect inguinal hernias.^[12]^ When incarceration occurs in an inguinal hernia, it causes an irreducible mass in the groin or scrotum, along with tenderness, redness, and swelling, accompanied by nausea and vomiting, as well as other symptoms. If not treated in time, serious complications, such as intestinal obstruction, intestinal strangulation, necrosis, or even testicular or ovarian necrosis, may occur.^[13,14]^ The traditional treatment of an incarcerated indirect inguinal hernia involves making an inguinal incision, locating the inguinal sac, releasing the internal ring orifice, removing the hernia contents, separating the hernia sac, and performing high ligation of the hernia sac. However, when an incarcerated inguinal hernia occurs, the tissue layers cannot be clearly distinguished because of the severe local edema caused by the incarcerated contents of the hernia. There is a higher chance of damage to the surrounding tissues, such as the spermatic vessels, vas deferens, inguinal nerves, and other local structures, during separation of the hernia sac, which may result in serious perioperative complications.^[15]^

Laparoscopic surgery for treatment of pediatric incarcerated indirect inguinal hernia has been reported both nationally and internationally.^[16,17]^ It has been suggested that laparoscopy is a safe and relatively less traumatic surgical option in the treatment of incarcerated indirect inguinal hernia in children. The laparoscopic surgery should be performed as soon as possible in cases of incarcerated hernias with ovarian content. Compared to traditional surgery, laparoscopic-assisted surgery for incarcerated inguinal hernia has more advantages. Based on our experience, the advantages of laparoscopic-assisted surgery are as followed: Under direct visualization of the laparoscopy, the contents of the hernia can be clearly visualized, which allows for convenient monitoring of these contents throughout the process of reduction. Following successful reduction, blood flow to the hernia contents can be observed to determine the presence of ischemic necrosis of the viscera (Fig. 1). In our study intestinal necrosis after reduction was observed in 2/36 patients, who consequently underwent intestinal resection and intestinal anastomosis. General anesthesia is helpful in relaxing the abdominal muscles and internal ring orifice, thereby giving rise to a higher success rate of manual reduction.^[1]^ Furthermore, pneumoperitoneum is helpful in the expansion of the deep ring orifice and the reduction of incarcerated contents.^[18]^ Among the 36 children in the observation group who received laparoscopic surgical treatment, 6 patients had spontaneous replacement of the incarcerated hernia after induction of general anesthesia, while the remaining 30 patients were successfully underwent smooth manual reduction after administration of general anesthesia, without the need for incision of the deep ring orifice. Among children under 2 years of age, as many as 56% of cases have contralateral patent processus vaginalis, but detecting the presence of these contralateral patent processus vaginalis is often difficult in traditional open surgery via inguinal incision.^[18]^ In contrast, during laparoscopic surgery the contralateral deep ring orifice can be easily observed and evaluated. In the observation group, 9/36 patients (25%) were found to have contralateral patent processus vaginalis in the abdominal cavity; hence, high ligation was performed bilaterally to prevent postoperative hernia occurrence (Fig. 2). In contrast, in the control group, 5 patients (22.7%) had postoperative contralateral inguinal hernia, and reoperation was performed after an interval of 3 months. Intraperitoneal treatment of an incarcerated indirect inguinal hernia can be performed laparoscopically without inguinal incision, even a few days after the onset of incarceration of an inguinal hernia;^[19,20]^ neither dissection of the inguinal canal nor excision and closure of the hernia sac via open surgery is necessary. In such cases, laparoscopy over open surgery can be effective in minimizing postoperative complications, such as groin or scrotal hematoma.

Although laparoscopic-assisted treatment of incarcerated indirect inguinal hernia in children has obvious advantages over traditional inguinal incision surgery, we believe that the following points pertaining to the operation should be considered: Strict observation of the indications for surgery, especially in children with severe dehydration on admission, which is always accompanied by severe systemic infection or obvious abdominal distention, since insertion of a trocar may be difficult and poses a risk of damaging the dilated bowel. Therefore, laparoscopic treatment was not recommended in such cases. The volume of the abdominal cavity of children is small; therefore, prior to the start of surgery, the buttocks on the affected side should be elevated and tilted to the healthy side by 15° in order to gain better exposure of the internal ring orifice on the affected side. Under direct vision of the laparoscope, the body surface projection site of the internal inguinal ring on the affected side can be determined, which is important for the insertion of the suture apparatus into the extraperitoneal space. To avoid injury, the suture needle should be inserted lateral to the inferior epigastric vein. In boys with incarcerated hernia, attention should be paid to the protection of the vas deferens and spermatic cord blood vessels, which should be withdrawn slowly after thread insertion, ensuring that the vas deferens and spermatic vessels are lateral to the thread (Fig. 3). The contents of incarcerated hernias in girls are often the ovary, which is always positioned at the opening of the internal inguinal ring after reduction. It is necessary to insert auxiliary forceps to pull up the ovary in order to completely expose the inner ring opening, and to confirm that there are no fallopian tubes or other organs and tissues within the hernia sac (Fig. 1). Simultaneous squeezing of the scrotum and tightening of the thread should be performed to remove gas accumulated in the scrotum after the surgery to prevent postoperative pneumatosis of the scrotum or groin. After tightening the suture knot, the skin of the abdominal wall was lifted to confirm that the knot was located in the extraperitoneal space. The knot was visible outside the inner ring during laparoscopic visualization. If the knot is placed in the subcutaneous fat layer, postoperative tissue reactions may occur, leading to infection. In children with secondary pus infection in the hernia sac, drainage tubes should be placed into the scrotum or groin before ligating the orifice of the inner ring. Closure of the inner-ring orifice should be reconfirmed, and the surrounding tissue should be checked for any damage. If no damage is visible, the laparoscope can be withdrawn and the abdomen closed.

In conclusion, laparoscopic-assisted treatment of incarcerated indirect inguinal hernias in pediatric patients is significantly superior to traditional open surgery in terms of overall procedure time, intraoperative blood loss, postoperative recovery time, and length of hospital stay. During the laparoscopic surgery, the blood flow to the incarcerated contents can be closely observed, the presence of organ or tissue injury or necrosis can be accurately assessed, patency of the contralateral processus vaginalis can be effectively detected, and any possible postoperative complications, such as occurrence of contralateral or homolateral hernia, as well as the need for reoperation, can be effectively prevented. Considering all the aforementioned points, laparoscopic-assisted hernia repair in pediatric patients is worth promoting for clinical application. However, further prospective studies, especially randomized clinical trials, are warranted before final recommendations are made.

## Author contributions

**Conceptualization:** Bowen Hu.

**Data curation:** Huanfei Ding, Fei Guo.

**Formal analysis:** Guofeng Zhang, Huanfei Ding.

**Investigation:** Huanfei Ding.

**Methodology:** Huanfei Ding, Di Wang.

**Resources:** Di Wang.

**Software:** Fei Guo.

**Supervision:** Bowen Hu.

**Validation:** Di Wang.

**Writing – original draft:** Guofeng Zhang.

**Writing – review & editing:** Fei Guo, Bowen Hu.
